# Regulation of the Brain Neural Niche by Soluble Molecule Akhirin

**DOI:** 10.3390/jdb9030029

**Published:** 2021-07-26

**Authors:** Mikiko Kudo, Kunimasa Ohta

**Affiliations:** Department of Stem Cell Biology, Faculty of Arts and Science, Kyushu University, 744 Motooka, Nishi-Ku, Fukuoka 819-0395, Japan; kudo.mikiko.490@s.kyushu-u.ac.jp

**Keywords:** Akhirin, neurogenesis, vasculogenesis, LCCL domain, vWF domain, hydrocephalus

## Abstract

In the central nervous system (CNS), which comprises the eyes, spinal cord, and brain, neural cells are produced by the repeated division of neural stem cells (NSCs) during the development of the CNS. Contrary to the notion that the CNS is relatively static with a limited cell turnover, cells with stem cell-like properties have been isolated from most neural tissues. The microenvironment, also known as the NSC niche, consists of NSCs/neural progenitor cells, other neurons, glial cells, and blood vessels; this niche is thought to regulate neurogenesis and the differentiation of NSCs into neurons and glia. Although it has been established that neurons, glia, and blood vessels interact with each other in a complex manner to generate neural tissues in the NSC niche, the underlying molecular mechanisms in the CNS niche are unclear. Herein, we would like to introduce the extracellular secreted protein, Akhirin (AKH; Akhi is the Bengali translation for eye). AKH is specifically expressed in the CNS niche—the ciliary body epithelium in the retina, the central canal of the spinal cord, the subventricular zone, and the subgranular zone of the dentate gyrus of the hippocampus—and is supposedly involved in NSC niche regulation. In this review, we discuss the role of AKH as a niche molecule during mouse brain formation.

## 1. Introduction

The central nervous system (CNS; eyes, spinal cord, and brain) acts as a controller that receives, sorts, and organizes information from all over the body and sends the corresponding commands, which is vital for survival. The CNS is formed by the repeated division and proliferation of neural stem cells (NSCs) and neural progenitor cells (NPCs). Dividing immature neurons differentiate into mature neurons. A hallmark of the NSCs/NPCs in the CNS is that they have extremely high proliferation during development; however, once the respective tissues are formed, their rate of cell division reduces dramatically. Therefore, it is important to understand when neuron production is active and how tissue formation progresses during tissue development.

Several studies have identified various cellular sources of NSCs in the adult vertebrate eye [1]. Retinal NSCs are present in the ciliary body epithelium [2,3], iris pigment epithelium [4], peripheral margin of the retina [5], and Müller cells [6,7]. Müller cells are radial glial cells, with morphology and expression of glial markers similar to those of embryonic radial cells, which are used as progenitor cells in the CNS. To date, it is believed that Müller cells are the endogenous NSCs in the retina.

In the spinal cord, several cell types have been identified in the central canal, including cuboidal, tanycytic, and radial classes of lumen-contacted ciliated ependymal cells [8]. Numerous studies have indicated that ependymal cells localized in the dorsal central canal, originated from radial glial cells, show NSC activity [9]. Ependymal cells also contribute to the regeneration of oligodendrocytes and remyelination after spinal cord injury [10].

In the adult mouse brain, several studies have indicated two major neurogenic niches: the subventricular zone (SVZ) lining the lateral ventricle and the subgranular zone (SGZ) of the dentate gyrus (DG) of the hippocampus in the CNS [11,12]. In the SVZ, type B stem cells give rise to type C transit-amplifying cells which, in turn, produce type A neuroblasts [13]. Type B and C cells form a tubular network through which type A neuroblasts migrate into the rostral migratory stream toward the olfactory bulbs. In the SGZ, proliferating radial and nonradial precursors give rise to intermediate progenitors which, in turn, generate neuroblasts. Immature neurons migrate into the inner granule cell layer and differentiate into dentate granule cells in the hippocampus [13].

NSCs/NPCs are constantly maintained in a specific microenvironment (niche) since the time of development processes and throughout adulthood [14]. Typically, equilibrium between cell proliferation and differentiation between the two cell populations, NSCs and NPCs, is important for CNS development. Neurons emerge from a pool of NSCs/NPCs through neurogenesis, which is regulated by many extrinsic and intrinsic factors. The niche in which NSCs are maintained consists of a complex array of other neurons, blood vessels, and other glial cells. The division and self-renewal of NSCs are regulated by specialized niche regulators secreted by these cells. Despite the relevance of the fate of NSCs/NPCs, which is ultimately reflected in the final number of newly generated neurons, the timing and number of divisions of NSCs and their differentiation into neurons are flexible processes; moreover, in several cases, not all types of intermediate progenitors are generated in a clonal lineage [15,16]. Thus, the mechanisms that control their development processes are poorly understood.

Herein, we introduce the secreted protein, Akhirin (AKH; Akhi is the Bengali translation for eye), which was isolated from embryonic day 6 (E6) chick lens using signal sequence trap cDNA screening [17]. We have previously reported extensive expression of AKH in the niches of the CNS. AKH is specifically expressed in the ciliary marginal zone of the retina [18], the middle and ventral central canal of the spinal cord [19], and the SVZ and the DG of the hippocampus in the mouse CNS [20]. As AKH exhibits heterophilic cell adhesion activity, which has been confirmed by cell aggregation analysis [18], it is supposed to function as an extracellular adhesion factor regulating these niches in the CNS. In this review, after summarizing the molecular structure of AKH and its role in the eye and spinal cord, we mainly discuss its role in the brain as a niche regulator in the CNS.

## 2. Possible Roles of the vWF-A and LCCL Domains in AKH

**Figure 1:**
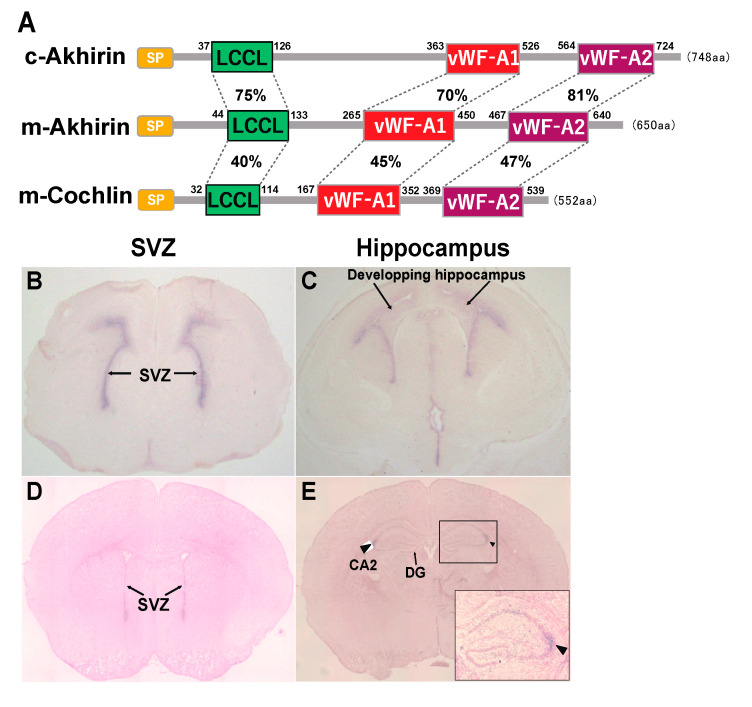
(**A**): A simple schematic diagram of chick and mouse AKH, mouse cochlin protein structure. The amino acid number is presented for each domain. (**B**–**E**): The expression of *AKH* mRNA in SVZ (**B**,**D**) and the hippocampus region (**C**,**E**) of the mouse brain at E17 (**B**,**C**) and P20 (**D**,**E**). Arrows indicate SVZ niche areas along the LV regions (**B**,**D**) and the developing hippocampus (**C**). Arrowheads indicate the hippocampal CA2 region (**E**).

The structure of AKH comprises two von Willebrand factor-A (vWF-A) domains and one Limulus factor C, Coch-5b2 and Lgl1 (LCCL) domain. The chick AKH has an open reading frame of 748 amino acid residues, and the mouse AKH has an open reading frame of 650 amino acid residues (Figure 1A). AKH has relatively high homology to vitrin [21] and cochlin [22]. In mice, AKH can be regarded as a factor almost identical to vitrin, and mouse cochlin in composed of 522 amino acid residues. Based on the two vWF-A domains in AKH, cell aggregation analysis using AKH-expressing transfectants indicated the role of AKH in cell adhesion [18]. As both the control cells and AKH-expressing transfectants adhered to immobilized AKH protein, we concluded that AKH has heterophilic cell adhesion activity [18]. It is one of great interest to identify the molecules that interact with AKH.

The vWF-A domain is involved in blood clotting. In vWF disease, the absence or malfunction of vWF-A results in bleeding which is difficult to control. Most of the vWF-A domains are synthesized in the endothelial cells of blood vessels. The vWF-A domain 1 directly binds to angiogenesis-inducing factors such as vascular endothelial growth factor-A (VEGF-A) and platelet-derived growth factor-BB (PDGF-BB). VEGF and PDGF-BB play an important role in vascularization [23] and neurogenesis, respectively, during early development [24].

Although the interaction of blood vessels with NSCs in the adult brain niche has already been widely reported [25,26,27], recent reports have shown that the timing of differentiation of NSCs/NPCs into neurons is implicated in the interaction between embryonic vascularization and neurogenesis. For instance, Di Marco et al. showed that nascent periventricular vessels interact with dividing apical neural progenitors by using vascular filopodia induced by the upregulation of VEGF-A in a cell-cycle-dependent manner [23]. They concluded that vascular filopodia helps in fine-tuning NSC behavior for proper brain development. As AKH contains two vWF-A domains, it is interesting to examine the molecular interactions between AKH and VEGF-A and PDGF-BB.

The LCCL domain is found in the biodefense factor C of the horseshoe crab, which is a lipopolysaccharide (LPS)-binding protein [28]. LPS is a constituent of the outer membrane of the cell wall of gram-negative bacteria. LPS can bind to Toll-like receptor 4 (TLR4), which is present on the surface of the host cell membrane and works as an endotoxin [29]. TLR family members are involved in the expression of proinflammatory cytokines and play an important role in innate immunity. Recent reports have shown that the LCCL domain is cleaved from the cochin protein, which sequesters infiltrating bacteria and protects hearing in the organ of Corti [28,30].

Neurons, immune system cells, and blood vessels form a tight network with each other to maintain immune homeostasis in the brain. The LCCL domain binds to LPS and plays a role in immune defense. Infections of bacteria may cause encephalitis, meningitis, and various other diseases in the brain and, occasionally, cause disturbances in the blood-brain barrier (between the neurons and vascular units) and the blood-cerebrospinal fluid barrier (BCSFB; between the cerebrospinal fluid (CSF) and choroid plexus (ChP)) [31]. The ChP is a vascular-rich tissue in the ventricle facing the NSC niche that produces and secretes CSF. Immune system activation following a bacterial infection in a pregnant mother increases the number of activated microglia in the ChP of the fetal brain, which disrupts the intercellular adhesion of ependymal cells, causing the disarrangement of the BCSFB and/or an increase in polarity, which biases the direction of the microglial neurites; this consequently results in abnormal cortical layer formation after birth [32,33]. The progression of layer formation is active during the embryonic and immediate postnatal periods. The neurons migrate toward the surface of the brain from the ventricular zones, forming the six-layered structure (inside-out). The ventricular zones consist of epithelial tissue, and the cell junctions are rigid. The disrupted regulation of cell junctions on the ventricular surface, especially cadherins, which are calcium (Ca^2+^)-dependent adhesion molecules between epithelial cells, causes abnormal layer formation [34]. In addition, since AKH has heterophilic cell adhesive activity [18], it is highly predictable that the loss of AKH will result in disruption of the tight junctions. Thus, it is likely that there is a mutual relationship between vascularization-neurogenesis and microglia during brain formation; however, the detailed molecular mechanism has not yet been elucidated. Therefore, the activation and neurite polarity of microglia may explain the cause of ventricle expansion in the *AKH*-/- mouse brain.

## 3. AKH Localizes in the Niche of the Eye

Different neuronal NSCs/NPCs exist in the vertebrate retina, and their proliferation and differentiation are influenced by a combination of intrinsic and extrinsic factors [35,36]. In chick peripheral retina, both *AKH* mRNA and protein are expressed through the ciliary epithelial layer in the embryonic stage. In the chick embryo, AKH expression is observed in the head ectoderm overlying the lens vesicle at stage 17 and in the retinal pigment epithelial layer at stage 22. Although AKH expression changes during the embryonic stage, AKH accumulates in the presumptive ciliary marginal zone at the postnatal stage where the NSCs/NPCs are localized [18]. As *AKH* mRNA and protein are co-localized in NSCs/NPCs, and AKH exhibits heterophilic cell adhesion activity, we hypothesized that AKH secreted from these cells is associated with other extracellular matrix components on their surface to regulate the niche [18]. Unfortunately, we could not observe the expression of AKH in E14 mouse retina.

## 4. AKH Localizes in the Niche of the Spinal Cord

The spinal cord is the caudal portion of the CNS and transduces information between the brain and the body. NSCs have been isolated from the ependymal zone surrounding the central canal of the spinal cord [37]. A recent study showed that NSCs are the most dorsally located glial fibrillary acid protein (GFAP)-positive cells lying ependymally [38]. AKH expression was observed in the spinal cord of mice on embryonic day 9.5 (E9.5), which disappeared by postnatal day 30 (P30). *AKH*-/- mice showed reduced spinal cord size compared to that in wild-type mice (*AKH+/+*). The expression patterns of ependymal niche molecules (nestin and GFAP) in *AKH*-/- mice were changed when compared with those of *AKH*+/+ mice in vivo [19]. In vitro culture of the spinal cord neurospheres showed significant reduction in the size of the neurospheres of *AKH*-/- mice compared with those of *AKH+/+* mice [19]. Interestingly, the distribution of ependymal proliferation factors (Cyclin D2 and vimentin) and proliferation markers (Ki67) in the neurospheres derived from *AKH*-/- was disturbed, indicating the involvement of AKH in NSCs/NPCs regulation. 

In general, ependymal cells of the spinal cord are normally quiescent in adult mice. However, when the spinal cord is damaged, ependymal cells are rapidly activated and undergo differentiation to form astrocytes at the injured site [8]. Although the expression of *AKH* in the central canal ependymal cells is very low or not observed in the central canal at P30, AKH expression is rapidly upregulated in ependymal cells after spinal cord injury, suggesting that AKH is involved in post-injury neuronal neogenesis [19]. These observations suggest that AKH plays a crucial role in spinal cord formation in mice by regulating the ependymal niche in the central canal [38].

## 5. AKH Is Exclusively Localized in Brain Neurogenic Niches

The adult mammalian brain contains billions of neurons assembled in defined neural circuits, which are the essential components for mediating the higher functions of the CNS. During the development of the mouse brain, NSCs exist around the ventricles, and the newborn neurons migrate to their destination through various pathways. In the niches, neurons and glia cells, such as microglia, astrocytes, and oligodendrocytes, emerge; they create a feedback interaction system via numerous secreted and contact-mediated signals for the regulation between quiescence and cell division of NSCs/NPCs. Although NSCs disappear from most parts of the brain after birth, they are still localized at the SVZ on the lateral wall of the LV and the SGZ of the hippocampal DG where they continue to produce neurons throughout life [38,39]. 

The expression of *AKH* at the SVZ was already observed at E17.5, which then disappeared by P20 (Figure 1B,D). In the hippocampal DG, *AKH* expression was observed in the entire hippocampal region immediately after birth, followed by a specific expression in the hippocampal CA2 region at P20 (Figure 1C,E). Thus, from the embryonic stage, AKH is expressed in the brain niche areas and disappears in tandem with the cessation of neurogenesis around P20 when brain formation is approximately complete; this observation suggests the involvement of AKH in neurogenesis and neuronal differentiation during early development but not in adult neurogenesis [20].

Newborn neurons migrate to other regions in the brain by forming special chain-like structures, which suggests that the interaction between newborn neurons and the extracellular matrix, such as AKH, is important in this process. NSCs are localized in the periventricular region, and nascent neurons migrate to the hippocampal region for hippocampal area formation. One possibility is that AKH might be involved in the migration of newborn neurons to the CA2 region because AKH secreted by NSCs is supposed to attach to the surface and interacts with the substrate during migration. The CA2 region of the hippocampus is a less well-characterized region than the CA1 and CA3 regions. In recent years, studies have reported the importance of the CA2 region in memory updating—a timeline of memory—and social memory [40,41,42]. When neuronal migration to the CA2 region is impaired due to the loss of AKH, psychiatric disorders, such as autism spectrum disorder, may occur. To examine the effect of AKH loss in the CA2 region, we are in the process of preparing a behavioral test battery with *AKH*-/- mice.

## 6. Effects of *AKH* Knockout on Neurogenesis and Neuronal Differentiation in the Brain NSC Niche

**Figure 2:**
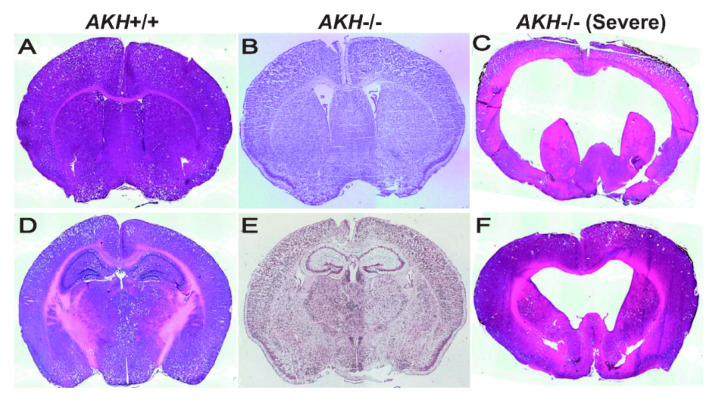
Histomorphology of the SVZ (**A**–**C**) and the hippocampal regions (**D**–**F**) in mouse brain on hematoxylin-eosin (HE) (**A**,**C**,**D**,**F**) and Nissl (**B**,**E**) staining. *AKH*+/+ (**A**,**D**), *AKH*-/- (**B**,**E**), and *AKH*-/- hydrocephalus-like mouse (**C**,**F**).

Self-renewal and differentiation potential of NSCs are dynamically regulated by various niche-derived factors, which relay signals in an autocrine or paracrine manner together with transcription factors that respond to those signals. To investigate whether AKH is a niche factor, we compared the brain morphology using *AKH*-/- and *AKH*+/+ mice and found that the ventricles of *AKH*-/- mice were widely expanded when compared with those of *AKH*+/+ mice (Figure 2A,B) [20]. The hippocampal DG region was reduced in *AKH*-/- mice when compared to that in *AKH*+/+ mice (Figure 2D,E) [20]. Furthermore, lower proportion of GFAP, SOX2, and Ki67 trip; e-positive (GFAP^+^/SOX2^+^/Ki67^+^) cells was observed in *AKH*-/-, indicating reduced NSC proliferation, but higher population of GFAP and SOX2 double-positive (GFAP^+^/SOX2^+^/Ki67^−^) cells was increased in *AKH*-/- mice, indicating increase of quiescent NSCs. Finally, *AKH* deficiency inhibited the differentiation of NSCs into mature neurons and reduced the length of their neurites [20]. These results suggest that the loss of *AKH* causes NSCs to lose their proliferative capacity and become quiescent, resulting in a decrease in neurogenesis from NSCs, leading to the enlargement of the ventricles and reduction of the DG area during early development.

## 7. Relationship between AKH and Hydrocephalus

Hydrocephalus is a frequent neurological disorder caused by the expansion of the cerebral ventricles and is associated with high morbidity and mortality rates [43]. Different forms of hydrocephalus have been identified: Noncommunicating hydrocephalus is caused by a blockage in the ventricular system, mainly at the aqueduct level between the third and fourth ventricles. In contrast, the ventricular system is not obstructed in communicating hydrocephalus. Although multiple genes and environmental factors are involved in hydrocephalus development, the molecular mechanisms underlying this condition remain unclear. Due to insufficient knowledge regarding the molecular basis of hydrocephalus, its clinical treatment is limited to invasive methods, with failure rates close to 50%.

Hydrocephalus-like enlargement of the ventricles was observed in the *AKH*-/- brain [20]. Interestingly, we found that approximately 3% of *AKH*-/- mice showed severely malformed brains that resembled a hydrocephalic brain morphology (Figure 2C,F). We were unable to analyze these fulminant *AKH*-/- mice in detail as these mutants died within the first month after birth. These phenotypes of *AKH*-/- hydrocephalic mice are similar to those of heterozygous *Nfib* and *Nfix* double mutant mice [44] and Yap mutant hydrocephalic mutant mice [45]. Although the ventricles expanded similarly to those in hydrocephalus because of these gene deletions, the molecular mechanisms underlying this condition remain unclear. Therefore, it is plausible to examine the molecular interactions between these genes.

## 8. Conclusions

**Figure 3:**
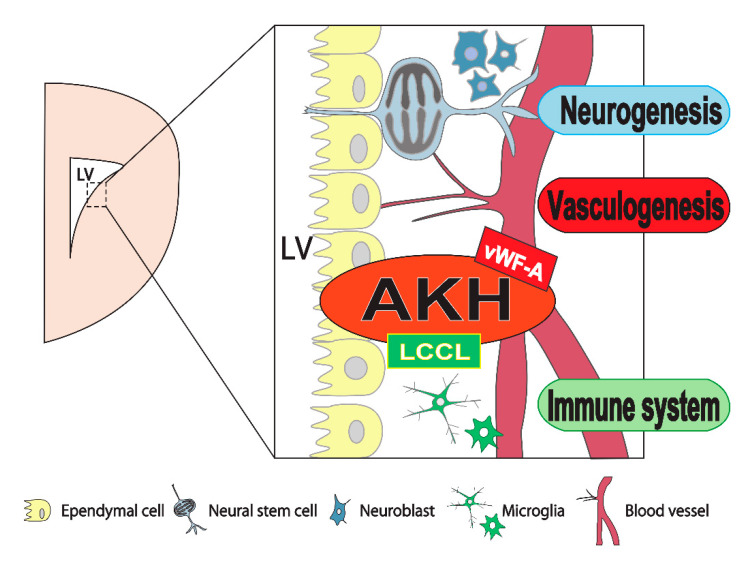
AKH is expressed in the neural stem cell niche and acts as a regulator. AKH is involved in the maintenance of neural stem cell proliferation, vasculogenesis, and the immune system during neurogenesis.

*AKH* deficiency inhibits the proliferation of NSCs, resulting in a decrease in the total number of neurons in the brain niche area, thereby resulting in ventricular expansion in patients with hydrocephalus. It is of interest to examine the human *AKH* gene sequence in patients with hydrocephalus in the future. At present, the physiological functions of AKH remain unclear. While we can predict the important functions of AKH during brain formation, such as neurogenesis, vasculogenesis, and immunocompetence, more studies are needed to clarify the functional consequences of the defects in brain formation in *AKH*-/- mice. To end this, it is necessary to determine the detailed expression and localization of AKH in developed mice brains, etc. Thus, we hope that a more detailed function of AKH will be discovered by analyzing the effect of *AKH* deficiency on the interaction between NSCs and blood vessels, and on microglia (Figure 3). To date, research in the fields of neurogenesis, vasculogenesis, and immunocompetence has been specialized and advanced. We propose that AKH is an interesting molecule that links these three fields and regulates brain formation.

## Data Availability

Not applicable.
